# Extremophilic Microorganisms for the Green Synthesis of Antibacterial Nanoparticles

**DOI:** 10.3390/microorganisms10101885

**Published:** 2022-09-21

**Authors:** Ida Romano, Giuseppe Vitiello, Noemi Gallucci, Rocco Di Girolamo, Andrea Cattaneo, Annarita Poli, Paola Di Donato

**Affiliations:** 1 Institute of Biomolecular Chemistry, National Research Council of Italy, Via Campi Flegrei, 34, 80078 Pozzuoli, Italy; 2 Department of Chemical, Materials and Production Engineering, University of Naples Federico II, Piazzale V. Tecchio 80, 80125 Naples, Italy; 3 CSGI (Center for Colloid and Surface Science), Via della Lastruccia 3, 50019 Sesto Fiorentino, Italy; 4 Department of Chemical Sciences, University of Naples Federico II, Via Cinthia 4, 80126 Naples, Italy; 5 Department of Science and Technology, University “Parthenope”, Centro Direzionale-Isola C4, 80143 Naples, Italy

**Keywords:** biogenic synthesis, extremophiles, metal nanoparticles, antibacterial activity

## Abstract

The biogenic synthesis of nanomaterials, i.e., synthesis carried out by means of living organisms, is an emerging technique in nanotechnology since it represents a greener and more eco-friendly method for the production of nanomaterials. In this line, in order to find new biological entities capable of biogenic synthesis, we tested the ability of some extremophilic microorganisms to carry out the biogenic production of AgNPs and SeNPs. Silver NPs were produced extracellularly by means of the thermophilic *Thermus thermophilus* strain SAMU; the haloalkaliphilic *Halomonas campaniensis* strain 5AG was instead found to be useful for the synthesis of SeNPs. The structural characterization of the biogenic nanoparticles showed that both the Ag and Se NPs possessed a protein coating on their surface and that they were organized in aggregates. Moreover, both types of NPs were found be able to exert an interesting antibacterial effect against either Gram-positive or Gram-negative species. This study confirmed that extremophilic microorganisms can be considered valuable producers of biologically active nanoparticles; nevertheless, further experiments must be performed to improve the synthesis protocols in addition to the downstream processes.

## 1. Introduction

Nanotechnology has emerged in recent years as an interdisciplinary technology that encompasses physics, chemistry, biology, biotechnology, material science, and medicine. Presented for the first time in a lecture titled “There’s plenty of room at the bottom” held by Richard Feynman at the American Institute of Technology in 1959, nanotechnology was described as a complex of technologies aiming at the production and the exploitation of objects or materials smaller than 1 mm. The prefix “nano” is a Greek word (*nanos*) that means “dwarf”; indeed, nanotechnology refers to materials defined as nanowires, nanofibers, nanoparticles (NPs), and so on, whose dimensions usually range from 1 to 100 nm in each spatial dimension. NPs can display different shapes; for example, they can be spherical, triangular, or rod-shaped, and, depending on the chemical nature of the starting material, they can be classified as carbon-based NPs, dendrimeric NPs, polymeric NPs, nanocomposites, and metal NPs [1,2]. NPs technology is an area of increasing interest; indeed, owing to their unique properties, NPs possess multiple functionalities making them useful for manifold applications. With particular regard to metal NPs such as gold, silver, titanium, zirconium, and selenium particles, they are exploited or are currently under investigation for their application in different fields including biotechnology, nanotechnology, physics, chemistry, medicine, and material science electronics. The synthesis of NPs can be carried out by either chemical or physical methods by means of two main approaches: the top-down strategy in which the bulk materials are broken into nanosized particles by means of pounding, chipping method, extruding, or ball milling [1,2], and the bottom-up approach in which NPs are produced starting from atoms or molecules that self-assemble to the final nanomolecular structures.

Nevertheless, the currently used methods of synthesis pose several concerns, such as the environmental impact, the toxicity of the involved reagents, and the formation of undesired by-products. Therefore, the need for the implementation of a more sustainable production of NPs has strongly pushed the search for green methods such as biogenic synthesis, i.e., nanoparticle synthesis carried out with a bottom-up approach by means of either uni- or multicellular organisms including bacteria, fungi, plants, and lichens [2,3]. Biogenic nanoparticles are considered greener and more environmentally friendly since biogenic synthesis requires no toxic chemicals and can be carried out at ambient conditions of both temperature and pressure. Biogenic synthesis is usually carried out by proteins that are produced either intra- or extracellularly by the biogenic entity; such proteins act by catalyzing the metals’ reduction and then modulate the NPs’ morphology [2]. Among the biological NPs producers, microbial species represent a valuable tool not only because they are easier to handle, but also because the downstream process for their recovery is simpler than that required by classical chemical methods [4].

The biogenic synthesis of metal NPs is, therefore, an object of increasing attention as a green strategy for the production of sustainable nanomaterials for biological and biotechnological applications [5,6]. Here, we focused our attention on two types of broadly used metal NPs that can be produced by using microbial species, i.e., Ag- and Se-based nanoparticles.

Silver nanoparticles (AgNPs) possess a wide array of biological properties, such as anticancer, antimicrobial, antibiofilm, and disinfectant properties; moreover, they find applications in the fields of diagnostics, in the construction of biosensors and conductive materials, and in optical applications such as metal-enhanced fluorescence and surface-enhanced Raman scattering [7,8,9]. On the other hand, Selenium nanoparticles (SeNPs) have gained attention due to their potential applications in different fields, such as medical diagnosis; for their anticancer properties—bare or conjugated with other anticancer agents; or, finally, as antibacterial agents [9,10].

The biological tools tested in this study included some extremophilic species. Extremophiles are a group of living organisms belonging to all the domains of life (*Archaea*, *Bacteria*, and *Eucarya*) that can survive and require extreme conditions in terms of pressure, temperature, ionic strength, and radiation; thus, their manifold properties make them the object of continual interest in different fields including ecology, astrobiology, industrial biotechnology, and renewable energy production [11,12]. Their ability to adapt to a variety of harsh conditions relies on their peculiar metabolism and on their capacity to produce biomolecules (lipids, osmolytes, and proteins) which allow to them to survive in extreme environments. Their potential to produce such peculiar molecules make them ideal bio-factories for the field of biotechnology. The species investigated include three thermophiles, i.e., those microbial species whose optimal growth temperature is in the range from 60 °C to 85 °C, and three haloalkaliphiles, i.e., microbial species that require high pH values and high salt concentrations to thrive. The biosynthesized Ag and SeNPs were characterized by means of UV–Vis spectroscopy, dynamic light scattering (DLS), transmission electron microscopy (TEM), Fourier transform infrared spectroscopy (FT-IR), and Zeta potential. Both types of nanoparticles were also tested for their antibacterial properties against *Escherichia coli* DSM 648 and *Kokuria rhizophila* DSM 348.

## 2. Materials and Methods

### 2.1. Bacterial Strain Culture Conditions

The following thermophilic microorganisms: *Thermus thermophilus* strain SAMU (SAMU) DSM 15284 [13]; *Sulfolobus sulfataricus* strain MT4 (MT4) ATCC 49155 [14]; *Parageobacillus thermantarcticus* strain M1 (M1) DSM 9572 [15], formerly *Bacillus thermantarcticus* [16]; and *Geobacillus thermantarcticus* [17], and the following haloalkaliphilic microorganisms: *Halomonas campaniensis* strain 5AG (5AG) DSM 15293 [18]; *Salinivibrio costicola* subsp. *alcaliphilus* strain 18M (18M) DSM 16359 [19]; and *Oceanobacillus oncorhynchi* subsp. *incaldanensis* strain 20AG (20AG) DSM 16557 [20], all present at the ICB-CNR cell bank collection, were grown at their optimal pH, temperature, and medium conditions, as previously reported [14,15,16,17,18,19,20]. The inoculum size was 5:100 *v*/*v* for the thermophilic species, while for the haloalkaliphilic ones, it was 4:100 *v*/*v*.

### 2.2. Selection of Strains Producing Silver Nanoparticles (AgNPs)

The thermophilic strains SAMU, MT4, and M1 were grown in 1000 mL Erlenmeyer flasks at 75 °C for 24 h, 85 °C for 72 h, and 60 °C for 24 h, respectively, as previously described. Cell-free culture supernatants (CFS), collected by centrifugation at 6500 rpm for 20 min at 4 °C, were used for nanoparticle synthesis. Optimization of AgNP synthesis was performed by testing the effect of the following reaction parameters: temperature at 50, 65, and 80 °C; CFS/distilled water ratio, 1:4 *v*/*v*, 2:3 *v*/*v*, and 4:1 *v*/*v*; and AgNO_3_ concentration, i.e., 0.5 mM and 1.0 mM. In all cases, all the reaction mixtures (total volume of 25 mL) were stirred at 120 rpm for 24 h, and aliquots were withdrawn after 1, 3, 6, and 24 h of incubation. Control experiments were carried out by incubating the uninoculated culture media containing silver nitrate and aqueous solutions of AgNO_3_ in the above-described conditions. AgNP production was monitored by observing color intensity changes during the incubation and by measuring the absorbance of reaction mixtures at 240 nm, as previously reported [21].

### 2.3. AgNP Production, Isolation, and Purification

The CFS (CFS/water ratio 2/3) from the strain SAMU was incubated at 80 °C in the presence of 0.5 mM AgNO_3_ for 24 h; after this time, the CFS was diluted with 1:1 *v*/*v* distilled water and the AgNPs were recovered by centrifugation at 17,000 rpm for 20 min at 4 °C (Beckman Coulter, AvantiTM J-20 XP Centrifuge, Brea, CA, USA). The AgNP pellet was washed 3 times with distilled water and then dispersed in 1 mL distilled water for the characterization and the antibacterial activity tests.

### 2.4. Selection of Strains Producing Selenium Nanoparticles (SeNPs)

For the selection of the SeNPs producers, the haloalkaliphilic strains 5AG, 18M, and 20AG were grown in 50 mL Falcon in the presence of Na_2_SeO_3_ (from 50 to 3200 ppm) in medium A, containing (g/L) yeast extract, 10, Na_3_citrate, 3; KCl, 2; MgSO_4_·7 H_2_O, 1; FeSO_4_, 0.050; NaCl, 100; Na_2_CO_3_, 3; and MnCl_2_ 4 H_2_O solution (0.36 g/L), 1 mL. The reaction was carried out at 36 °C and 200 rpm for 24 h; aliquots (2 mL) were withdrawn after 1, 3, 6, and 24 h of incubation. Control experiments were carried out by incubating the uninoculated culture media containing sodium selenite and aqueous solutions of Na_2_SeO_3_ in the above-described conditions. SeNP production was monitored by the visual control of color changes in the reaction mixtures (from transparent/pale yellow to red), while cell growth was assessed by measuring turbidity at λ 600 nm, as previously reported [18,19,20]. The results were normalized against the turbidity values measured by growing the strains in the absence of selenite.

### 2.5. SeNP Synthesis, Isolation, and Purification

Cells of the haloalkaliphilic strain 5AG (inoculum size 1:50) were incubated in 200 mL of the previously described medium A, in medium B (containing (g/L) K_2_HPO_4_, 7; KH_2_PO_4_, 2; MgSO_4_·7H_2_O, 0.1; (NH_4_)_2_SO_4_, 1; Na_2_CO_3_, 3; NaCl, 100; and 500 µL/L of biotin solution, 100 mg/L, 1% glucose), and in medium C (displaying the same composition as that of medium B except for glucose that was replaced by 15 g/L sterilized molasses). The reaction was carried out in the presence of 200 ppm Na_2_SeO_3_ at 36 °C, under stirring at 200 rpm for 24 h in the case of medium A, whereas, in the case of media B and C, this was prolonged for 72 h. Cells were harvested by centrifugation at 6500 rpm for 20′ at 4 °C (Beckman Coulter, AvantiTM J-14 Centrifuge), washed three times with 60 mL minimal medium B depleted of glucose, and then re-suspended in 10 mL of distilled water. SeNPs were recovered by lysis of cells after treatment in an autoclave at 121 °C for 20 min. Cell lysates were centrifuged at 3000 rpm for 20 min at 4 °C (Beckman Coulter, AvantiTM J-20 XP Centrifuge); the resulting pellets were washed three times with distilled water and the supernatants were collected, filtered on a 0.20 μm filter, and sonicated for 10 min (ULTRA SONIC NDI 104 H). NPs were recovered by freeze-drying of the collected supernatants. The lyophilized samples were suspended in 1 mL distilled water for characterization and antibacterial activity tests.

### 2.6. Characterization of Nanoparticles

#### 2.6.1. UV–Vis Spectroscopy

The absorption properties of the nanoparticles were investigated by a Unico UV-2100 spectrophotometer (Agilent, Palo Alto, CA, USA) at a resolution of 5 nm, in the range of 200–800 nm. The NPs were dispersed in distilled water, and the UV–Vis spectra of the sodium selenite and silver nitrate solutions were taken as controls.

#### 2.6.2. Fourier Transform Infrared Spectroscopy (FT-IR)

The FT-IR spectra were registered by mixing NP samples (2–3 mg) mixed with KBr and taken under a pressure of 200 bar for 10 min. The KBr pellet containing the sample was then analyzed by means of an FT-IR spectrophotometer model: Perkin-Elmer Mod. Gx.

#### 2.6.3. Transmission Electron Microscopy (TEM)

The morphology of the biogenic NPs was investigated by means of an EI TECNAI G2 200 kV (Fei Company, Dawson Creek Drive Hillsboro, Hillsborough, CA, USA) microscope. Images were taken by placing a drop of NP samples (previously sonicated for 10 min with an ULTRA SONIC NDI 104 H bath at 25 °C) on a carbon-coated copper grid, which were allowed to air-dry before imaging.

#### 2.6.4. Dynamic Light Scattering (DLS)

The particle size distributions of the NPs were determined with a home-made instrument composed of a Photocor compact goniometer (Moscow, Russia), an SMD 6000 Laser Quantum 50 mW light source (Laser Quantum, Fremont, CA, USA) operating at 532.5 Å, a photomultiplier (PMT-120-OP/B), and a correlator (Flex02−01D) from Correlator.com (accessed on 1 January 2016) (Shenzhen, China). The experiments were carried out at the constant temperature of 25.0 ± 0.1 °C using a thermostatic bath and at the scattering angle θ of 90°. The NP samples (50 mg of lyophilized sample in 1 mL of distilled water) were previously sonicated for 10′ with an ULTRA SONIC NDI 104 H bath at 25 °C. The scattered intensity correlation function was analyzed using a regularization algorithm [22]. The diffusion coefficient of each population of diffusing particles was calculated as the z-average of the diffusion coefficients of the corresponding distributions [23,24,25]. The culture media used for NP biosynthesis were taken as blanks.

#### 2.6.5. Zeta Potential

The ζ-potential measurements were performed to assess the electrostatic properties of the NPs. Lyophilized samples (50 mg in 1 mL distilled water, previously sonicated for 10′ with an ULTRA SONIC NDI 104 H bath at 25 °C) were analyzed by means of electrophoretic light scattering using a Zetasizer Nano ZSP (Malvern Instruments, Malvern, UK). Each measurement was recorded at 25 °C upon a 30 s equilibration time and the average of three measurements at a stationary level was taken. The ζ-potential was calculated using the Smoluchowski model.

#### 2.6.6. Nanoparticle Antibacterial Activity Assay

The antibacterial activity of the biosynthesized NPs was examined against the Gram-negative *Escherichia coli* DSM 648 and the Gram-positive *Kokuria rhizophila* DSM 348 (both purchased from DSMZ) by means of the well diffusion method, measuring the zone of inhibition. For both microorganisms, a single colony was inoculated in 10 mL of the specific growth medium, i.e., LB for *E. coli* and NB for *K. rhizophila* (LB: yeast extract 5 g/L, Triptone 10 g/L, and NaCl 10 g/L in distilled water, and NB: lemco powder 0.2% *w*/*v*, yeast extract 0.2% *w*/*v*, Peptone 0.5% *w*/*v*, and NaCl 0.5% *w*/*v* in distilled water); after 24 h at 36 °C, cell suspensions were diluted up to 10^−5^ CFU/m, and 100 μL of this suspension was seeded on agar plates supplemented with the growth media, LB and NB for *E. coli* and *K. rhizophila*, respectively. Sterile discs containing water solutions of the AgNPs (with total amounts of 0.04 mg, 0.08 mg, and 0.16 mg) and of the SeNPs (with total amounts of 10.0 mg, 20.0 mg, and 40.0 mg), obtained as previously described, were put on the agar plates’ surfaces, and the inhibition halos were measured after 24 h at 36 °C.

## 3. Results

### 3.1. Selection of NP Producer Strains

#### 3.1.1. Selection of AgNP Producer Strains and Biosynthesis Optimization

The biosynthesis of silver nanoparticles was carried out extracellularly using the cell-free supernatants (CFS) recovered by the cellular biomass growth of the thermophilic strains SAMU, MT4, and M1. In order to select either the best microbial producer or the optimal biosynthesis conditions, the effect of the following parameters was tested: incubation time, temperature, CFS dilution, and AgNO_3_ concentration.

**Figure 1:**
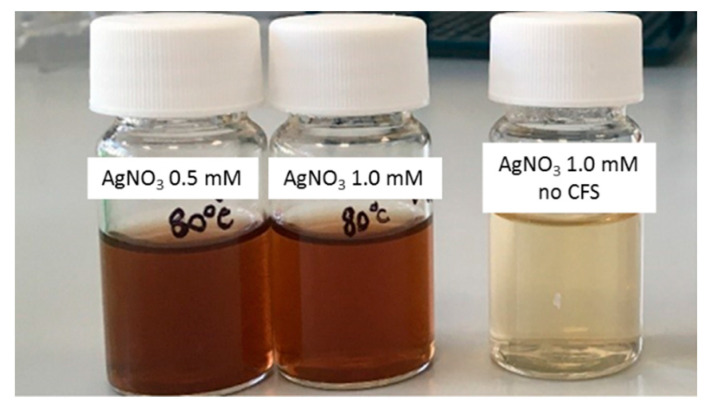
AgNP formation by the SAMU strain. The reduction of silver nitrate, evidenced by color change of the reaction mixture from light to dark brown, is indicative of NP production. For comparison, on the right, a nitrate solution without CFS is shown.

For all the experiments, the AgNP production was followed by a visual inspection of the reaction solutions: the appearance of a brownish color (Figure 1) was indicative of nanoparticle generation.

**Figure 2:**
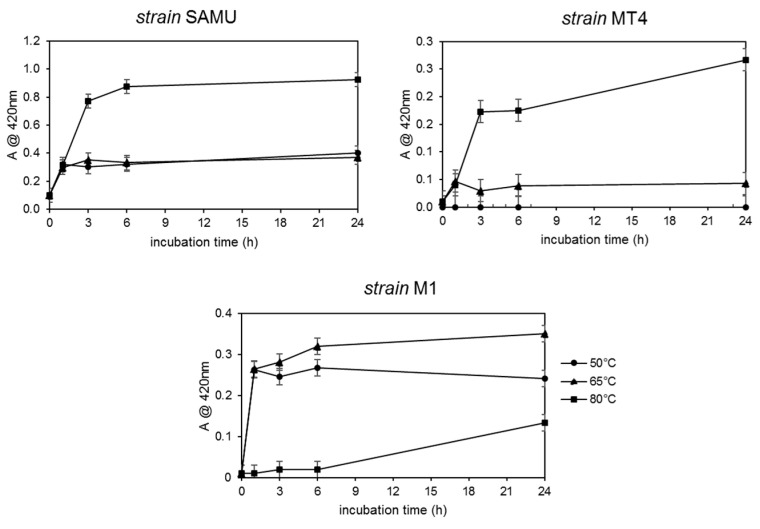
AgNPs yields’ dependence on temperature and time of incubation. The formation of silver NPs was monitored by measuring the solutions’ absorbance at 420 nm.

The AgNP formation’s yield was measured by absorbance at 420 nm as previously described for their biogenic synthesis [21], and it was used to assess all the parameters’ effect; control experiments were carried out in which the AgNO_3_ was incubated in the absence of the CFS under the different conditions of temperature and nitrate concentrations. Initial experiments were performed to assess the optimal temperature conditions; synthesis was carried out at 50, 65, and 80 °C using a CFS/water 10/15 *v*/*v* ratio in the presence of 0.5 mM AgNO_3_. The reaction was monitored by measuring the absorbance at 420 nm (the λ value typical of the AgNPs) after 1, 3, 6, and 24 h of incubation. The results obtained are shown in Figure 2.

For both the SAMU and MT4 strains, the production of AgNPs increased with increasing incubation time and temperature: the highest AgNPs production (approximately twice that of the other strains) was reached after 24 h of reaction at 80 °C using the CFS from the SAMU species. In the case of the M1 strain, a similar trend regarding the incubation time was observed only at 50 °C and 65 °C; indeed, at 80 °C, no appreciable amounts of particles could be detected.

On the basis of these results, the cell-free supernatant from the SAMU strain was selected for the further experiments aiming at the optimization of the other synthesis parameters, i.e., the CFS dilution factor and AgNO_3_ concentration.

**Figure 3:**
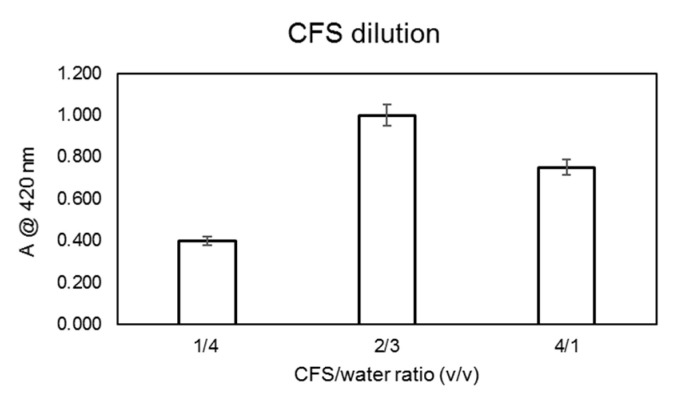
AgNPs yields’ dependence on the CFS/water dilution ratio. Experiments were carried out using the CFS from the SAMU strain at 80 °C and after 24 h of incubation. The formation of silver NPs was monitored by measuring the solutions’ absorbance at 420 nm.

For the optimization of the CFS dilution factor, the reaction was carried out at 80 °C for 14 h and in the presence of 0.5 mM AgNO_3_; the dilution factors assayed and the results obtained in terms of absorbance at 420 nm are shown in Figure 3.

**Figure 4:**
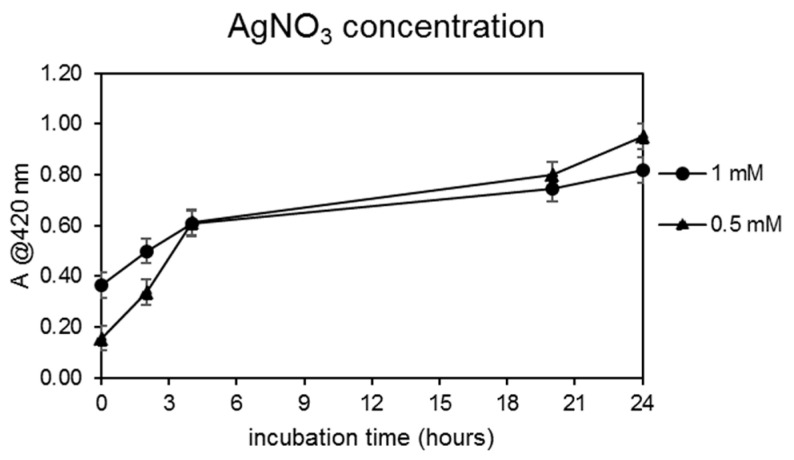
AgNPs yields’ dependence on the AgNO_3_ concentration. Experiments were carried out using the CFS from the SAMU strain (CFS/water ratio of 2/3) at 80 °C and for 24 h of incubation. The formation of silver NPs was monitored by measuring the solutions’ absorbance at 420 nm.

Finally, since the best CFS/water ratio in terms of AgNPs yield was found to be 2/3 *v*/*v*, the optimization of the AgNO_3_ concentration was carried out by incubating the CFS at this dilution ratio at 80 °C for 24 h in the presence of two different concentrations of silver nitrate, i.e., 0.5 and 1.0 mM. The results obtained are shown in Figure 4.

The results obtained highlighted that the best reaction conditions for AgNP biogenic synthesis were T = 80 °C, CFS/water ratio 2/3, t = 24 h, and 0.5 mM AgNO_3_. These reaction conditions were then used to synthesize AgNPs that were isolated and purified as described in Section 2.3 and which were subsequently subjected to characterization and biological activity assays.

#### 3.1.2. Selection of SeNP Producer Strains and Biosynthesis Optimization

**Figure 5:**
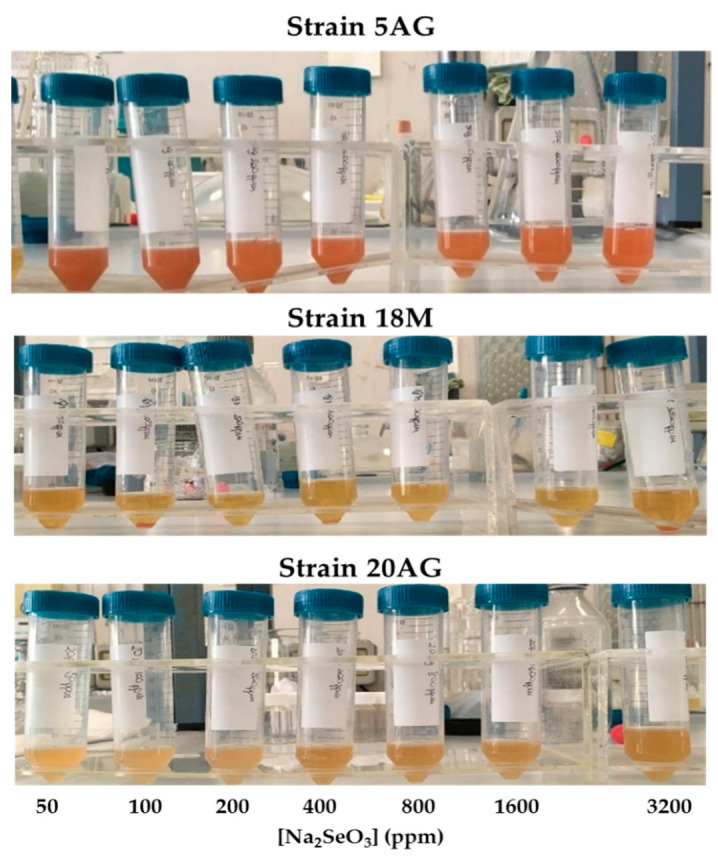
Selenite reduction by the haloalkaliphilic strains 5AG, 18M, and 20AG grown in medium A. The reduction of selenium (IV) to selenium (0) is evidenced by the appearance of the red color.

The screening of selenium nanoparticle producers was carried out using the cell cultures of the haloalkaliphilic strains 5AG, 18M, and 20AG whose ability to tolerate and reduce sodium selenite was tested on medium A in the presence of selenite concentrations ranging from 50 ppm to 3200 ppm. The ability of the haloalkaliphiles to catalyze selenite’s reduction was monitored by visual inspection of reaction solutions; the appearance of a reddish color (Figure 5) was indicative of its reduction for nanoparticle generation. Figure 5, where the obtained reaction mixtures are shown, suggests that at all the selenite concentrations tested, the 5AG strain was the most effective species for the reduction of selenite and, therefore, potentially for the biosynthesis of SeNPs, as compared with the 18M and 20AG strains.

**Figure 6:**
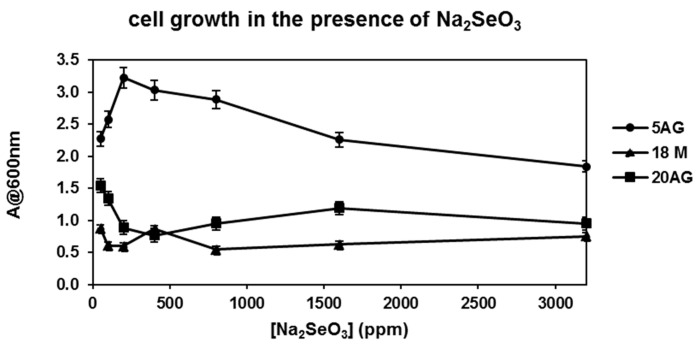
Haloalkaliphilic strain growth in medium A in the presence of Na_2_SeO_3_. Experiments were carried out at 36 °C and 200 rpm for 24 h. The cellular growth was monitored by measuring the solutions’ optical density at 600 nm.

In parallel, the cellular growth ability of all the strains in the presence of selenite was monitored by measuring the optical density of the incubation mixtures at 600 nm. The results are reported in Figure 6 where the optical density measured after 24 h incubation is reported as a function of the Na_2_SeO_3_ concentration.

As reported in Figure 6, the 5AG strain afforded a level of cellular growth higher than that of the other strains at all the selenite concentrations tested; therefore, this strain was selected for the biosynthesis of SeNPs. Since the highest cellular growth of 5AG was obtained in the presence of 200 ppm Na_2_SeO_3_, the production of nanoparticles was carried out by incubating the 5AG strain with this selenite concentration at 36 °C and 200 rpm for 24 h.

**Figure 7:**
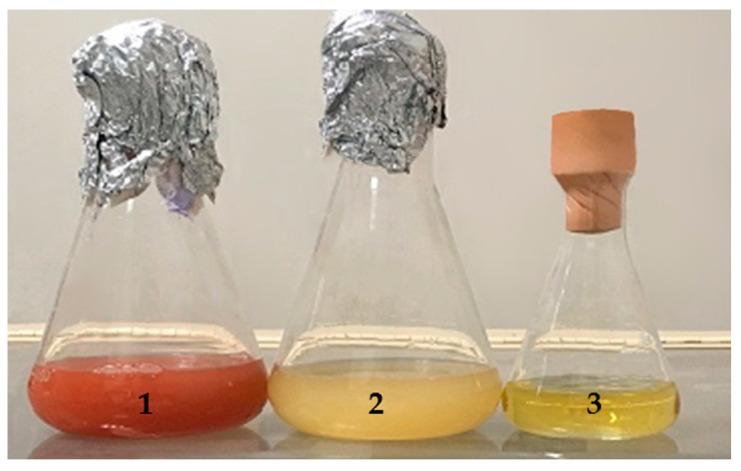
Selenite reduction by the haloalkaliphilic 5AG strain grown in medium A at 36 °C for 24 h and 200 rpm. Flask 1: The 5AG strain grown in the presence of 200 ppm Na_2_SeO_3_. Flask 2: The 5AG strain grown in the absence of Na_2_SeO_3_. Flask 3: Total of 200 ppm Na_2_SeO_3_ incubated in medium A.

Control experiments were carried out by incubating the 5AG strain in the absence of selenite and by incubating selenite in medium A, but in the absence of the strain. As shown in Figure 7, a visual inspection showed that, after 24 h at 36 °C and 200 rpm, the formation of a reddish could be detected only for the mixture containing both 200 ppm Na_2_SeO_3_ and the 5AG cells; on the contrary, when the strain was incubated with no selenite (flask 2 in Figure 7), or when selenite was incubated without the microorganisms (flask 3 in Figure 7), no formation of a reddish coloration could be detected.

**Figure 8:**
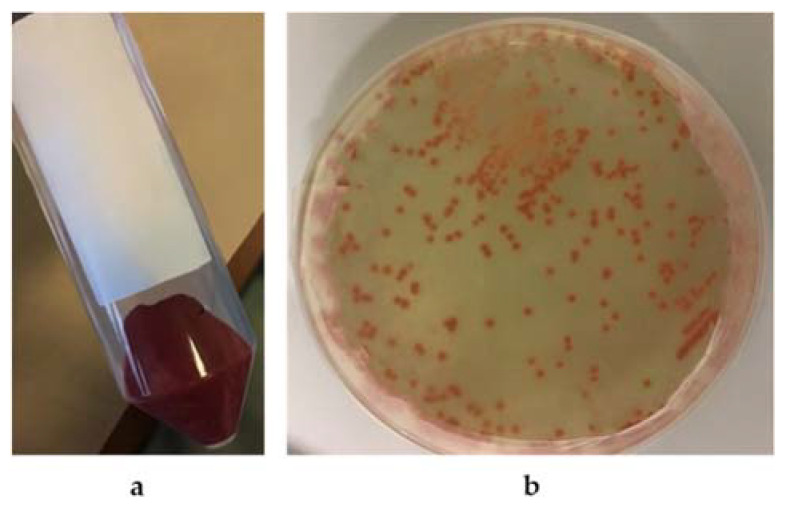
The 5AG strain’s cellular localization of selenite reduction products in medium A. Panel (**a**) cells incubated in liquid medium; Panel (**b**) cells incubated in solid medium.

The biogenic synthesis of SeNPs by the 5AG strain was probably an intracellular process, as already described for other biogenic systems [10,26]. Nevertheless, it cannot be excluded that the biosynthesis could also have taken place on the cells’ surface. After centrifugation of the incubation mixture, as shown in Figure 8a, the red color was retained by the cells, whereas the cell-free supernatant did not show any red coloration. A similar result was obtained by growing the 5AG strain in the solid medium A; as shown in Figure 8b, the red color was found only in association with the cells, which could be compatible either with intracellular synthesis or with NP production closely associated to the cells’ membrane.

For the optimization of the synthesis protocol, the effect of the chemical composition of the growth medium was also tested. Therefore, further experiments were carried out in the above-described conditions using the minimal medium B, containing glucose as a carbon source, and the minimal medium C which had the same chemical composition as that of medium B, but in which the carbon source was represented by a waste material recovered from food processing, i.e., molasses.

**Figure 9:**
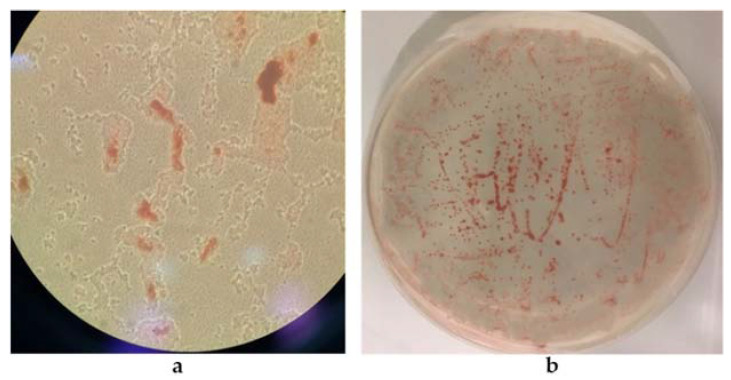
The 5AG strain’s cellular localization of selenite reduction products in medium B. Panel (**a**) optical microscopy (magnification 40×) of cells incubated in liquid medium; Panel (**b**) cells incubated in solid medium.

In medium B, the SeNP biosynthesis was a process that took place intracellularly or closely associated with the cell surface, as shown in Figure 9, where cells grown in either the liquid (Figure 9a) or solid (Figure 9b) medium retained the red color indicative of the selenite reduction products.

**Figure 10:**
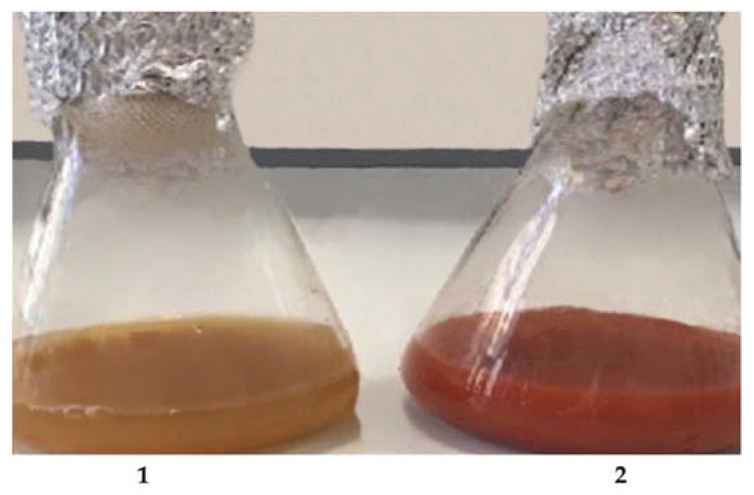
Selenite reduction by the 5AG strain grown in medium C, supplemented with molasses, at 36 °C for 24 h and 200 rpm. Flask 1: The 5AG strain grown in the absence of Na_2_SeO_3_. Flask 2: The 5AG strain grown in the presence of 200 ppm Na_2_SeO_3_.

The third medium tested, i.e., medium C, is a minimal medium containing molasses, a food industry residue, as the sole carbon source. Interestingly, the waste-based medium was also able to sustain either 5AG growth or its ability to reduce Na_2_SeO_3_. Control experiments were carried out by incubating the 5AG strain both in the absence and in the presence of sodium selenite in medium C. As shown in Figure 10, when cells were grown in liquid medium C without selenite (flask 1 in Figure 10), no red color was found; on the other hand, cells grown in the presence of Na_2_SeO_3_ (flask 2 in Figure 10) developed an intense red coloration, confirming the formation of the selenite reduction products.

The complex of these results highlighted that the best reaction conditions for SeNP biogenic synthesis were T = 36 °C, t = 24 h, 200 rpm agitation, and 200 ppm Na_2_SeO_3_. These reaction conditions were then used to synthesize SeNPs in all three media, i.e., media A, B, and C. The nanoparticles obtained in the three different media were isolated and purified as described in Section 2.5 and were subsequently subjected to characterization and to biological activity assays.

### 3.2. AgNPs’ and SeNPs’ Physicochemical Characterization

#### 3.2.1. Silver Nanoparticles (AgNPs)

**Figure 11:**
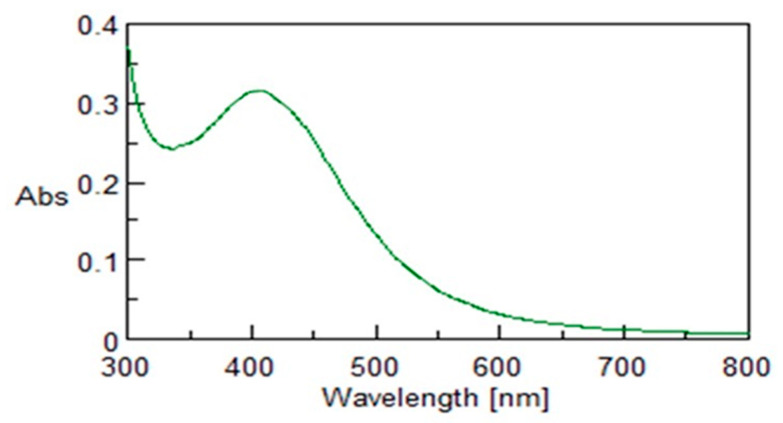
UV–Vis spectrum of AgNPs produced in the following conditions: CFS from SAMU/water ratio 2/3, T = 80 °C, and 24 h of incubation.

The obtained nanoparticles were firstly characterized by means of UV–Vis spectroscopy. The experimental spectrum showed a broad absorption band centered at approximately 420 nm (Figure 11), which is a typical feature previously reported for silver nanoparticles obtained from other biological sources [21], confirming their formation under the adopted synthesis conditions.

**Figure 12:**
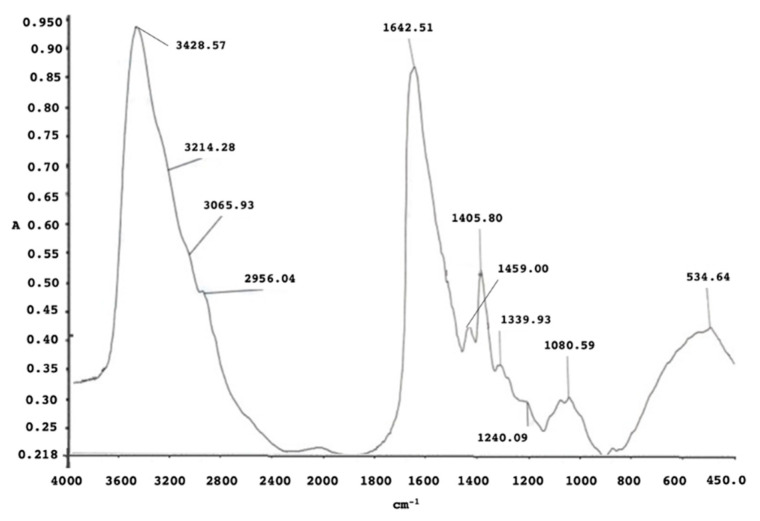
FT-IR spectrum of AgNPs produced by SAMU.

The FT-IR analysis (Figure 12) showed the main functional groups on the surface of the nanoparticles. Indeed, the main signals were found in the region of the N-H of amide I and II bands at 3428.5 cm^−1^ and 3214.2 cm^−1^; other signals ascribable to the amide bands were found at 1642 cm^−1^ and 1125 cm^−1^ related to vibration modes of the C=O of the carboxylic groups, and at 1460 cm^−1^ related to the stretching of the N-H group. These bands clearly suggested the presence of organic moieties, such as proteins and/or peptides, covering the NPs’ surface as a result of their formation and growth in the biological environment.

**Figure 13:**
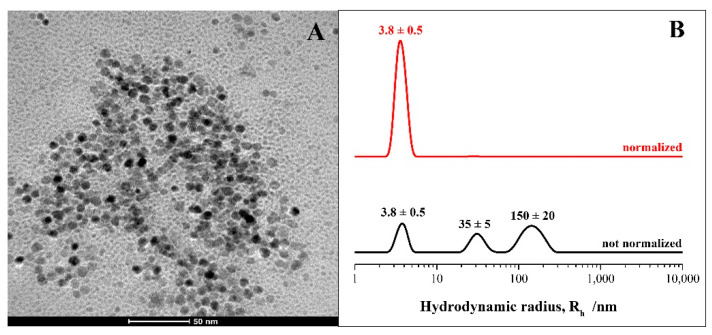
Panel (**A**) TEM micrographs (scale bar: 50 nm) and panel (**B**) hydrodynamic radius distributions of the AgNPs produced by SAMU.

A morphological analysis was carried out by acquiring TEM images, which gave us information about the shape and the size of AgNPs produced by the SAMU strain. As shown in Figure 13A, very small spherical nanoparticles with an average diameter of ~10 nm were obtained. DLS measurements were recorded to analyze the nanoparticles’ size distribution and to investigate the behavior of the obtained nanoparticles in an aqueous environment. From a quantitative point of view, the non-normalized hydrodynamic radius distribution of the AgNPs (Figure 13B, black line) indicated a polydisperse system with the presence of three populations. The first was centered at 3.8 nm and corresponded to bare nanoparticles; this represented the main population, as confirmed by the DLS curve normalized for molecular weight and as reported in Figure 13B (red line). The two other populations were centered at approximately 35 and 150 nm, respectively, and were probably ascribable to the presence of NP clusters of a larger size. In particular, their formation was induced by organic molecules, i.e., proteins, comprising the biological environment that wrapped the bare AgNPs, as also evidenced by the FT-IR analysis.

Then, the measurement of the Zeta potential provided information about the nanoparticles’ surface charge and colloidal stability since the potential’s magnitude directly correlated with the electrostatic repulsions, i.e., a high potential magnitude corresponded to high repulsion and, therefore, to high NP stability. The Zeta potential values of the AgNPs in the aqueous solution showed a value of −15 ± 1 mV, thus suggesting a low colloidal stability, as compared with the reference values reported in Table 1 [27]. Indeed, due to this low potential which was close to charge neutrality, the obtained AgNPs were widely characterized by weak electrostatic repulsions in the solution, which favored fast self-aggregation and precipitation phenomena.

#### 3.2.2. Selenium Nanoparticles (SeNPs)

**Figure 14:**
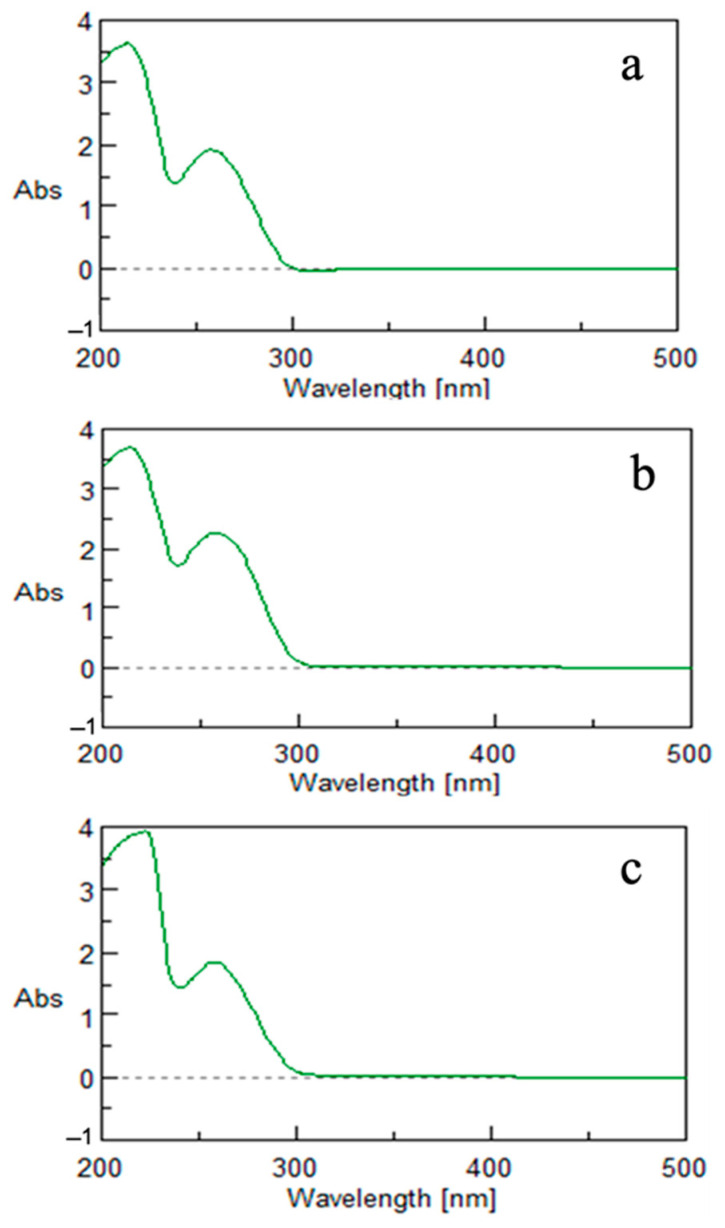
UV–Vis spectra of SeNPs produced in medium A (panel (**a**)), medium B (panel (**b**)), and waste-based medium C (panel (**c**)).

The obtained nanoparticles were first characterized by means of UV–Vis spectroscopy (Figure 14). The experimental spectrum showed a broad absorption band centered at approximately 265 nm, which was the typical feature previously reported for selenium nanoparticles obtained from other biological sources [28,29], confirming their formation by means of all the growth media used, i.e., the complex medium A (Figure 14a), the minimal medium B containing glucose as carbon source (Figure 14b), and, finally, the minimal medium C supplemented with waste molasses as a carbon source (Figure 14c).

**Figure 15:**
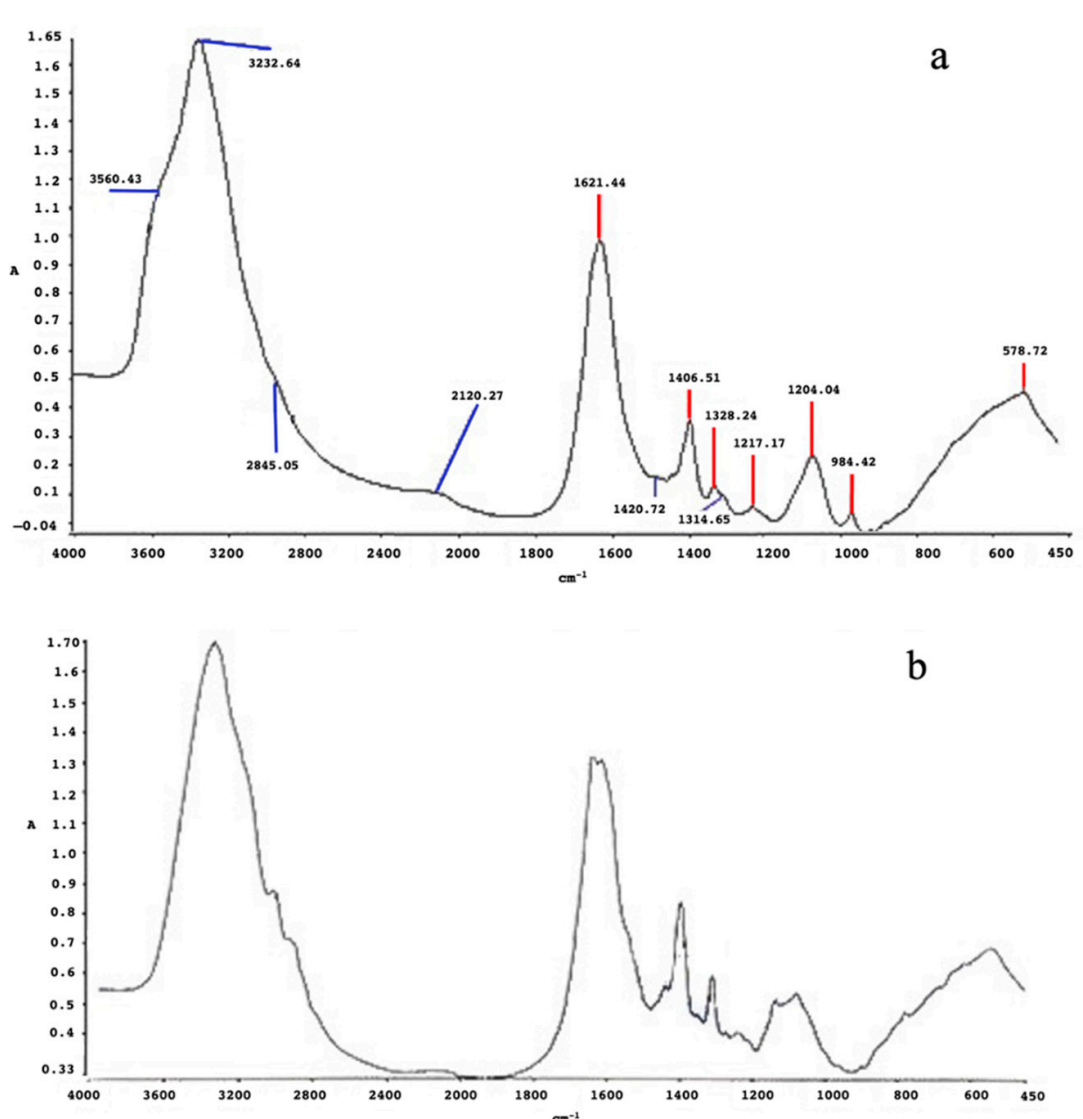
FT-IR spectra of SeNPs produced by 5AG in minimal medium B (panel (**a**)) and in minimal medium C (panel (**b**)).

The FT-IR analysis of the main functional groups on the surface of the SeNPs obtained from the 5AG strain was performed for the particles obtained in both the minimal culture media (i.e., medium B containing glucose and medium C containing molasses). Similar spectra for the SeNPs produced in both conditions were obtained (Figure 15, panels a and b, respectively), presenting a broad band in the 3200–3500 cm^−1^ range corresponding to the N-H stretching of amide I and II groups; a signal at approximately 1650 cm^−1^, which was related to vibration modes of the C=O of the carboxylic groups and to the bending of the N-H group; and a peak at 1460 cm^−1^, which was related to C-H bending. The presence of these peaks clearly indicated that, in this case also, the SeNPs were coated by biological molecules, such as proteins and metabolites. Indeed, the carboxyl groups of amino acid residues were able to stably bind selenium, suggesting that proteins (and other biomolecules) can exert a double role as both templating and stabilizing agents in an aqueous medium [30,31]. At the same time, the carbonyl groups of carbohydrates in the bacterial culture medium were responsible for Se^4+^ ion reduction when they bound to the SeNPs’ surface, thus driving their formation [32].

**Figure 16:**
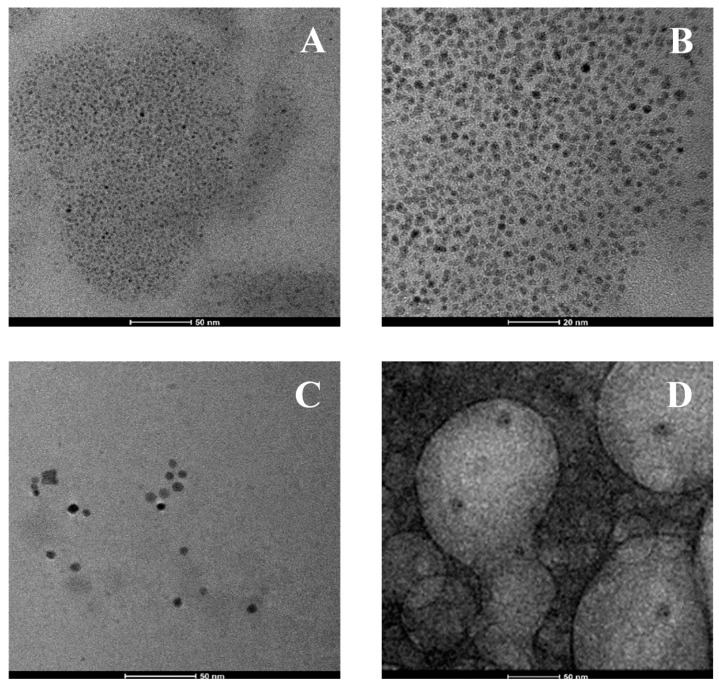
TEM micrographs of SeNPs produced by the 5AG strain in the complex matrix, scale bar: 50 nm and 20 nm (panels (**A**,**B**)); the glucose matrix, scale bar: 50 nm (panel (**C**)); and the molasses matrix, scale bar: 50 nm (panel (**D**)).

A morphological analysis was carried out by acquiring TEM images, which provided information about the shape and the size of the SeNPs formed by the 5AG bacterial strain on different bacterial matrices. In particular, very small spherical nanoparticles with average diameters of ~3 nm formed in the complex medium (Figure 16A,B), resulting in their being wrapped by an organic matrix (see Figure 16A) composed of the cytosol proteins in which the SeNPs remained dispersed. Instead, spherical nanoparticles with average diameters of ~10 nm formed in the glucose medium; these were not coated by any organic shell and did not create any aggregates (Figure 16C). In the molasses medium, despite the presence of large aggregates due to the self-aggregation of the cellular organic components, small SeNPs of approximately 5–10 nm in size were observed (Figure 16D).

**Figure 17:**
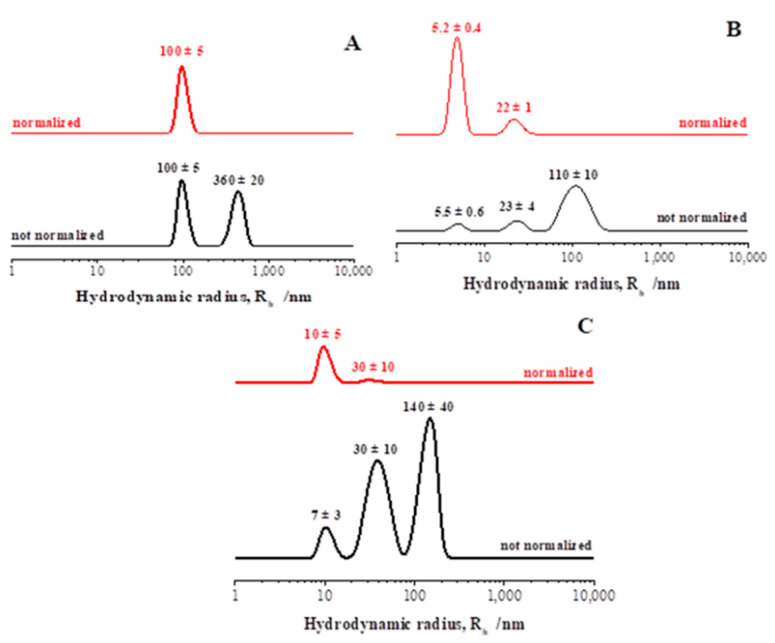
Hydrodynamic radius distributions of SeNPs produced by the 5AG strain in the complex medium A (panel (**A**)), the medium with glucose B (panel (**B**)), and the minimal medium with molasses C (panel (**C**)).

The size distribution and colloidal behavior of the obtained SeNPs in an aqueous environment was investigated by DLS analysis (Figure 17). All the samples were polydispersed, indicating the presence of different NP populations as shown by the non-normalized hydrodynamic radius distributions (Figure 17A–C, black lines). In all the cases, the presence of NP clusters induced by the presence of an organic matrix of biomolecules was observed. This evidence was also confirmed by the DLS curves normalized for the molecular weight (Figure 17A–C, red lines). However, in the case of the complex medium, a single population of approximately 100 nm in radius was observed (Figure 17A), which can be clearly ascribable to the self-aggregation of the primary SeNPs observed by TEM. In contrast, in the case of both the glucose and molasses media, two populations of smaller clusters of approximately 5–10 nm and 20–30 nm in radii, respectively, were obtained (Figure 17B,C). This could be due to the greater tendency of the proteins and metabolites of the cellular medium to organize themselves into organic aggregates (as shown in the TEM images), thus limiting their interaction with the NPs’ surfaces and the growth of NP clusters. Finally, the obtained SeNPs showed a ζ-potential value of −8 ± 1 mV, which confirmed their weak instability in an aqueous environment as already observed for the AgNPs.

### 3.3. Antibacterial Properties of AgNPs and SeNPs

The antibacterial properties of the nanoparticles were assayed as previously reported for other biogenic NPs by means of the well diffusion method [33,34]. Metal nanoparticles produced by the thermophilic SAMU strain and by the haloalkaliphilic 5AG strain were both assayed for their potential antibacterial properties against two known bacterial pathogens, i.e., *Escherichia coli* DSM 648 and *Kokuria rhizophila* DSM 348.

For the assay of the antimicrobial activity of the AgNPs produced by the SAMU strain, lyophilized nanoparticles were suspended in distilled water and the effect of different amounts were tested. The corresponding activity was quantified as inhibition halos, and the results are summarized in Table 2.

The AgNPs showed to be active against both the Gram-negative species *E. coli* and the Gram-positive species *K. rhizophila* with a very similar inhibitory effect for all the tested quantities.

**Figure 18:**
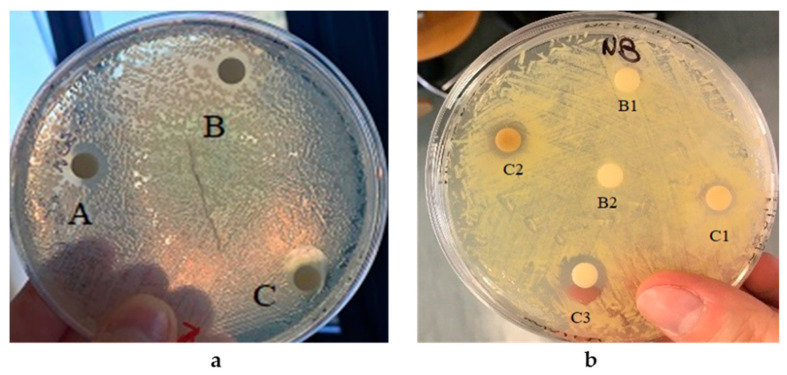
AgNPs’ effect against *E. coli* and *K. rhizophila.* Panel (**a**) *E. coli* in the presence of a AgNp solution containing 0.04 mg (spot B), 0.08 mg (spot A), or 0.16 mg (spot C). Panel (**b**) *K. rhizophila* in the presence of a AgNp solution containing 0.04 mg (spot C1), 0.08 mg (spot C2), or 0.16 mg (spot C3). Controls: 40 μL distilled water (spot B1) and CFS without AgNPs (spot B2).

The assay of the antimicrobial activity of the biogenic SeNPs by the 5AG strain was also carried out by suspending lyophilized nanoparticles in distilled water; the resulting solutions were seeded on the solid media (see Figure 18) in the presence of the two microbial pathogens already used for AgNPs. Additionally, in this case, the corresponding activity was quantified as inhibition halos whose values are reported in Table 3.

**Figure 19:**
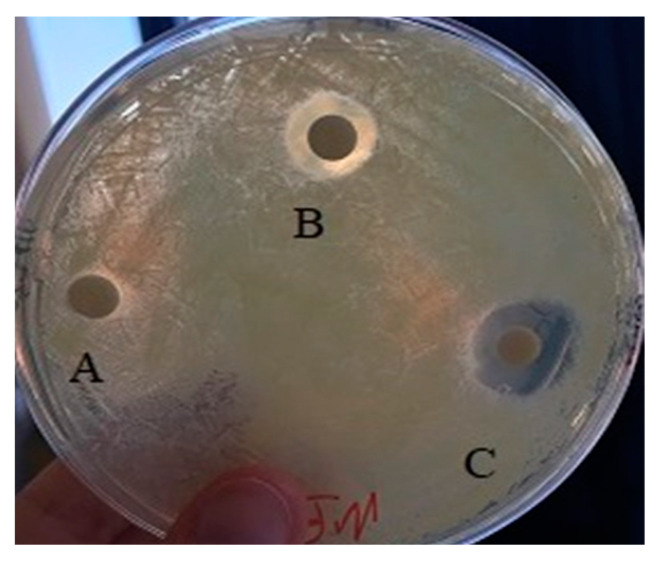
SeNPs’ effect against *K. rhizophila.* Inhibition halos obtained in the presence of 40 mg SeNP solution from medium A (spot A), from medium B (spot B), and from medium C (spot C).

Despite no appreciable antibacterial activity against *E. coli*, all the biogenic selenium NPs were found to exert an interesting antimicrobial effect against the Gram-positive species *K. rhizophila* (Figure 19). Moreover, the SeNPs produced using the waste-based medium, i.e., medium C supplemented with molasses, apparently showed the highest inhibitory effect.

## 4. Discussion

The biogenic synthesis of nanoparticles, i.e., NP production mediated by a biological entity such as microbial species, is an emerging field within green chemistry and nano-biotechnology. Indeed, biogenic synthesis, in contrast with the chemical and/or physical approaches, is a more eco-friendly and cost-effective strategy for obtaining nanoparticles since no toxic chemicals or high energy inputs are needed. In this regard, the search for microbial species able to carry out the biosynthesis of metal nanoparticles is a key issue for the development of the sustainable production of biotechnologically useful nanomaterials. The objective of this study was to find new microbial species to exploit as bio-factories for metal nanoparticle synthesis. Therefore, attention was paid to two main groups of extremophiles: thermophiles, which are microorganisms able to live at high temperatures, and haloalkaliphiles, which are microorganisms that require high pH and salt concentrations to thrive. Among the thermophilic species tested, the best AgNP producer was found to be the species *Thermus thermophilus* strain SAMU, which, to the best of our knowledge, is the first species from the genus Thermus to be described as a silver nanoparticle producer. Initial experiments were carried out to optimize the synthesis protocol. By increasing the temperature values and incubation time, the highest NP yields were obtained. Other parameters that were tested included the cell-free supernatant dilution factor and the silver nitrate concentration. The best results were obtained when using an intermediate value of the CFS/water ratio, whereas doubling the silver nitrate concentration (from 0.5 to 1.0 mM) did not significantly affect the NPs’ production yield. The AgNPs produced by the SAMU strain showed interesting physicochemical and biological properties. The structural characterization analysis evidenced the production of two main types of spherical NPs: bare ones, which were the most abundant, and protein-capped NPs, whose production was induced by the proteins that were present in the CFS. The antibacterial assay showed a good effectiveness of the AgNPs against either the Gram-positive or the Gram-negative pathogens tested. With regard to the SeNPs, in this case also, the first experiments were aimed at finding the most Se-tolerant microorganisms since selenium and its oxyanions, such as selenite, exert toxic effects on living organisms. Nevertheless, some microorganisms are able to catalyze selenium reduction with the consequent production of nanoparticles. Among the tested haloalkaliphiles, the species that showed the highest tolerance was *Halomonas campaniensis* strain 5AG. Several halophilic species have been described as metal NP producers; nevertheless, very few examples of species from the genus Halomonas [35] have previously been described as selenium nanoparticle producers. *H. campaniensis* was found to be able to tolerate selenite concentrations up to 3200 ppm in all the tested growth media. The biosynthesis of SeNPs by *H. campaniensis* was tested using either a complex growth medium or two minimal media in which glucose and a food waste, i.e., molasses, were used as the sole carbon sources. Interestingly, *H. campaniensis* retained its ability to catalyze the synthesis of SeNPs in the minimal medium supplemented with molasses. The structural characterization analysis showed that the type of growth medium used for the biosynthesis influenced the size of the SeNPs; indeed, while larger nanoparticles were formed in the complex medium, smaller populations of NPs were formed in the presence of other types of minimal media. In all the cases, the presence of protein coatings promoted the formation of aggregates. The three types of SeNPs obtained in this study also showed promising biological properties as potential antibacterial agents against the Gram-positive pathogen tested, whereas no appreciable effects could be detected on the Gram-negative strain. Additionally, in this case, the growth medium used for the biogenic synthesis affected the properties of the SeNPs; indeed, the NPs obtained on the molasses-containing medium showed higher antibacterial activity as compared with the other two types of nanoparticles. Such a result opens the way to a more sustainable strategy for the green synthesis of selenium nanoparticles since it can be carried out using a less expensive growth medium as compared with the classical complex ones based on commercially available nutrients.

In conclusion, the biogenic synthesis of metal nanoparticles is a green strategy for the production of biotechnologically useful nanomaterials. In this regard, extremophiles provide a valuable tool for obtaining antibacterial NPs that can be produced using inexpensive growth media (such as food-waste-based media), thus providing a greener and less expensive strategy for obtaining useful nanoparticles.

## Figures and Tables

**Table 1:** Nanoparticles’ stability as a function of ζ -potential: reference values and intervals.

NPs’ Stability	Zeta Potential (mV)
Rapid aggregation	From 0 to ±5
Weak stability	From ±10 to ±30
Moderate stability	From ±30 to ±40
Good stability	From ±40 to ±60
Excellent stability	≥±61

**Table 2:** Antimicrobial activity of AgNPs against *E. coli* and *K. rhizophila*.

Strain	AgNPs (mg)	Inhibition Halos (cm)
	0.04	0.7
*E. coli*	0.08	1.1
	0.16	1.3
	0.04	0.7
*K. rhizophila*	0.08	1.0
	0.16	1.2

**Table 3:** Antimicrobial activity of SeNPs against *K. rhizophila*.

	Inhibition Halos (cm)			
Strain	NPs (mg)	Medium A	Medium B	Medium C
*K. rhizophila*	10.0	0.5	0.6	0.7
	20.0	0.9	0.7	1.0
	40.0	1.2	1.0	1.5

## Data Availability

Not applicable.
